# Transcriptome and Hormone Analysis Revealed Jasmonic Acid-Mediated Immune Responses of Potato (*Solanum tuberosum*) to Potato Spindle Tuber Viroid Infection

**DOI:** 10.3390/antiox15010086

**Published:** 2026-01-08

**Authors:** Iva Marković, Bernard Jarić, Jana Oklešťková, Jitka Široká, Kristina Majsec, Jasna Milanović, Snježana Kereša, Ivanka Habuš Jerčić, Ondřej Novák, Snježana Mihaljević

**Affiliations:** 1 Division of Molecular Biology, Ruđer Bošković Institute, Bijenička Cesta 54, 10000 Zagreb, Croatiabernard.jaric@irb.hr (B.J.);; 2 Laboratory of Growth Regulators, Faculty of Science, Palacký University & Institute of Experimental Botany, Czech Academy of Sciences, Šlechtitelů 27, 77900 Olomouc, Czech Republic; jana.oklestkova@upol.cz (J.O.); ondrej.novak@upol.cz (O.N.); 3 Centre for Plant Protection, Croatian Agency for Agriculture and Food, Gorice 68b, 10000 Zagreb, Croatia; jasna.milanovic@hapih.hr; 4 Division of Plant Science, Faculty of Agriculture, University of Zagreb, Svetošimunska Cesta 25, 10000 Zagreb, Croatia; skeresa@agr.hr (S.K.); ihabus@agr.hr (I.H.J.)

**Keywords:** antioxidant responses, basal defense responses, biotic stress, growth–defense trade-off, hormone crosstalk, jasmonic acid, MeJA, potato, PSTVd, RNA-Seq

## Abstract

Potato is a globally important non-cereal crop in which infection with potato spindle tuber viroid (PSTVd) can cause stunted growth and significantly reduce tuber yield. We previously showed that PSTVd induces accumulation of the plant hormone jasmonic acid (JA) and alters antioxidant responses in potato plants. To clarify the role of JA in response to PSTVd, we analyzed disease development in transgenic JA-deficient *opr3* and JA-insensitive *coi1* lines compared to the wild-type. Transcriptomic analysis using RNA-Seq revealed that most genotype-specific differentially expressed genes (DEGs) in all comparisons were enriched in plant hormone signal transduction, plant-pathogen interaction, and MAPK signaling pathways, although the number of DEGs varied. These differences were confirmed by independent data from RT-qPCR, hormone, and hydrogen peroxide (H_2_O_2_) analyses. After PSTVd infection, *opr3* plants showed enhanced JA signaling and increased abscisic acid (ABA) and auxin (AUX) content. In contrast, *coi1* plants showed reduced ABA, AUX, and salicylic acid content. Both *opr3* and *coi1* plants showed reduced JA and H_2_O_2_ content and lower expression of defense-related genes, resulting in milder symptoms but increased viroid accumulation. In addition, treatment with methyl jasmonate alleviated symptoms in infected wild-type plants. Together, these results indicate a modulatory role for JA and JA signaling in basal immune responses and symptom development in the potato-PSTVd interaction.

## 1. Introduction

Potato (*Solanum tuberosum*) is the fourth most important food crop worldwide, after rice, wheat, and maize [1]. Potato spindle tuber viroid (PSTVd) is a subviral plant pathogen that can cause significant losses in yield and marketable quality of potato tubers. This threat is heightened by the fact that no potato varieties are known to be naturally resistant to this pathogen, and no curative treatment exists for this disease [2].

PSTVd is a small, non-coding, highly structured, circular, single-stranded RNA molecule consisting of approximately 341–364 nucleotides [3]. It is not clear how plants recognize this non-coding biotrophic pathogen, but transcriptomic studies on some viroid-host interactions suggest that viroids can activate pattern-triggered immunity (PTI) and effector-triggered immunity (ETI), as well as general downstream defense responses, including the generation of reactive oxygen species (ROS), mitogen-activated protein kinase (MAPK) cascades, and hormone signaling, which ultimately lead to transcriptional reprogramming of cells [3,4].

The plant hormone jasmonic acid (JA) is generally considered to play a defensive role in plant responses to necrotrophic pathogens, while salicylic acid (SA) is involved in responses to biotrophic pathogens [5,6]. However, increasing evidence supports a positive role for JA and its signaling pathway in defense against certain biotrophic fungal, bacterial, and viral pathogens in various plant species [7,8,9,10,11,12]. The accumulation of JA during pathogen perception promotes the expression of CORONATINE INSENSITIVE1 (COI1), which binds jasmonate ZIM domain proteins (JAZ) and promotes their degradation. This enables positive-acting transcription factors such as MYC2 to activate the transcription of JA-responsive genes involved in a wide range of defense responses, including maintenance of redox homeostasis, modulation of signaling crosstalk, and stimulation of defense compound production [13,14,15]. In response to pathogens, JA does not act alone but interacts with other hormones and signaling pathways through the actions of JAZ and MYC2 regulatory proteins [14,15].

Transcriptomic data from plant hosts infected with viroids indicate that the expression of genes involved in JA biosynthesis and signaling pathways is affected, depending on the specific viroid-host interaction [16,17,18,19,20,21,22,23]. However, studies examining whether changes in gene expression coincide with altered accumulation of endogenous JA levels in affected host plants are rare [24,25]. The accumulation of JA in potato leaves during PSTVd infection is also significant because there is little or no accumulation of SA [25], another important defense hormone that, along with JA, determines the outcome of defense responses. To elucidate the molecular mechanisms underlying basal defense responses in potato, this study analyzed the role of JA accumulation in response to PSTVd. We conducted a comparative transcriptomic analysis during PSTVd infection in transgenic lines with downregulated JA biosynthesis or JA signaling and in the corresponding wild-type potato using RNA-Seq. Transcriptome analysis showed that PSTVd infection suppresses the expression of genes related to photosynthesis and primary metabolism while activating genes involved in protein metabolism, signal transduction pathways, and defense responses. In this study, we focused on hormone, MAPK, and ROS signaling pathways. We also analyzed the temporal dynamics of gene expression, hormone levels, and H_2_O_2_ accumulation to determine how the timing of their expression influences plant susceptibility to PSTVd infection. Furthermore, we tested the effect of exogenous methyl jasmonate (MeJA) on gene expression and the perception of viroid infection. Key genes and pathways involved in basal defense against PSTVd in potato were identified. Results show that JA and its signaling modulate many defense responses to PSTVd in potato, and that their absence increases viroid accumulation but reduces symptom development, revealing new aspects of plant-viroid interactions.

## 2. Materials and Methods

### 2.1. Plant Material, Viroid Inoculation, and Sampling

Potato cv. Désirée wild-type, two transgenic *StOPR3*-RNAi silenced lines (*opr3*A3 and *opr3*Z2) resulting in JA deficiency, and two transgenic *StCOI1*-RNAi silenced lines (*coi1*H1 and *coi1*X5) resulting in JA insensitivity [26], were propagated in stem node tissue culture. The in vitro plantlets were transferred to a soil/perlite mixture (3:1) and grown in a phytochamber at 22 °C, 60% relative humidity, and a 16-h photoperiod with a light intensity of 160 μmol m^−2^ s^−1^. Healthy potato plants with 3–4 leaves were inoculated with H_2_O (mock) or a viroid inoculum (5.7 × 10^7^ viroid copies/μL) prepared from plants infected with PSTVd (GenBank: KF418768) as described [25]. Briefly, the two lower leaves were dusted with Celite 545 (Sigma-Aldrich, St. Louis, MO, USA) and rubbed with 2 µL of inoculum per leaf. After 10 min, the leaves were thoroughly washed with H_2_O. After inoculation, plants were grown at 26 °C to enhance viroid replication and symptom development [16]. The first three intact leaves above the inoculated leaves were harvested weekly from 1 to 8 weeks post-inoculation (wpi), immediately frozen in liquid nitrogen, and stored at −80 °C for RNA, hormone, and H_2_O_2_ quantification. Leaves from at least four mock- and PSTVd-inoculated plants per genotype per time point were harvested. Each biological replicate consisted of leaves from a single plant. The experiment was repeated twice.

For exogenous MeJA treatment, plants were sprayed twice with H_2_O or 1 mM MeJA (Sigma-Aldrich, St. Louis, MO, USA): one day before and six days after viroid inoculation (i.e., 24 h before the first sampling). Leaf samples from at least four mock- and PSTVd-inoculated plants per genotype per treatment per time point were harvested weekly from 1 to 6 wpi.

To distinguish effects of viroid inoculation from possible developmentally regulated changes, healthy, mock-inoculated plants served as controls in all experiments. The appearance of symptoms, such as epinasty and pointed upper leaves, was assessed visually, while growth retardation was evaluated by measuring plant height and fresh weight of the apical leaves (Figure S1).

### 2.2. RNA Extraction and RT-qPCR

Total RNA was extracted using the Direct-zol RNA Mini Prep Kit (Zymo Research, Irvine, CA, USA) according to the manufacturer’s instructions. Absolute quantification of PSTVd RNA was performed using the Brilliant III Ultra-Fast qRT-PCR Master Mix (Agilent, Santa Clara, CA, USA) in a duplex reaction with specific primers and probes for PSTVd and *cytochrome oxidase 1* (*COX*) gene as described [27,28].

For plant gene expression analysis, total RNA was reverse transcribed using the SuperScript IV First-Strand Synthesis System (Thermo Fisher Scientific, Waltham, MA, USA). qPCR was performed with the SsoAdvanced Universal SYBR Green Supermix (Bio-Rad, Hercules, CA, USA) in a CFX96 Touch Real-Time PCR Detection System (Bio-Rad, Hercules, CA, USA). The PCR cycling conditions were 95 °C for 30 s, followed by 40 cycles of 10 s at 95 °C and 20 s at 60 °C, then a dissociation curve was generated to verify amplification specificity. Data were normalized using an inter-plate calibrator to correct for run-to-run variability. Relative gene expression was calculated using the 2^−ΔΔCT^ method [29], with *elongation factor 1* (*EFα1*) as the reference gene [30]. Primers were designed using Primer3Plus version 3.3.0 or as described in [31,32,33,34,35] (Table S1). Primer efficiency and melt curves were checked and found acceptable for RT-qPCR.

### 2.3. RNA-Seq Gene Expression Analysis

Total RNA from three independent biological replicates (each an independent plant) per genotype and treatment was used to construct cDNA libraries with the TruSeq Stranded mRNA Library Prep Kit (Illumina, San Diego, CA, USA). Sequencing was performed by Macrogen (Seoul, South Korea) using paired-end sequencing on an Illumina NovaSeq 6000 platform. Quality control, adapter trimming, read filtering, mapping, and analysis were performed by Macrogen through their internal pipeline. Reads with mean Phred values below 15 and lengths shorter than 36 bp were removed. Quality statistics are shown in Table S2. Paired-end RNA-Seq reads were mapped to the reference genome SolTub_3.0 [36] with HISAT2 (v2.1.0) using the Bowtie2 (v2.3.4.1) aligner [37]. Mapped reads were assembled with StringTie to generate read counts, which were further analyzed in R with DESeq2 [38]. In DESeq2, size factors were estimated and used to normalize the libraries with the Relative Log Expression method. The negative binomial Wald test was applied to the normalized count data to determine *p*-values for differentially expressed genes (DEGs) between each comparison pair. Macrogen reported expression data with FDR-adjusted *p*-values for all comparisons. The criteria for DEGs were |log_2 _ fold change| ≥ 2 and *p*-value < 0.05. Gene ontology (GO) enrichment analysis was performed using g:Profiler (https://biit.cs.ut.ee/gprofiler/gost (accessed on 12 August 2022)), and data were visualized using ChiPlot (https://www.chiplot.online/polarPlot/circle_enrich_plot.html (accessed on 11 January 2025)). MapMan analysis was performed as described [39]. Venn diagrams and Kyoto Encyclopedia of Genes and Genomes (KEGG) analysis were generated using R packages (v4.2.2), including VennDiagram and KEGGREST. Transcription factor (TF) analysis was performed using PlantTFDB v5.0 [40].

### 2.4. Plant Hormone Analysis

The acidic plant hormones (SA; jasmonoyl-L-isoleucine, JA-Ile; *cis*-(+)-12-oxo-phytodienoic acid, *cis*-OPDA; abscisic acid, ABA; indole-3-acetic acid, IAA) were determined as described [41]. Briefly, samples (10 mg fresh weight) were extracted in cold 10% aqueous methanol with the addition of internal standards (IS) labeled with stable isotopes (20 pmol SA-*d*_4_, Sigma Aldrich, St. Louis, MO, USA; 5 pmol (−)-JA-*d*_2_-Ile, 10 pmol OPDA-*d*_5_ and 10 pmol IAA-^13^C_6_ OlChemIm Ltd., Olomouc, Czech Republic; 10 pmol ABA-*d*_6_, National Research Council Canada, Saskatoon, SK, Canada). The extracts were purified on Oasis^®^ HLB solid phase extraction columns (1 cc/30 mg, Waters) as described [42]. Analysis was performed on an Agilent 6490 Triple Quadrupole LC/MS system coupled to a 1290 Infinity LC system (Agilent Technologies, Santa Clara, CA, USA). The amount of SA-*d_4_* is set higher than that of other IS due to chromatographic issues related to peak shape (peak broadening associated with the history of the analytical column) and detection sensitivity. A level of 20 pmol ensures well-detectable chromatographic peaks of SA-*d_4_* in most matrices analyzed. Additionally, the higher SA-*d_4_* peak areas more closely match those of endogenous SA, which is important for accurate quantification. In contrast, the amount of (−)-JA-*d_2_*-Ile is set lower than that of other IS, as 5 pmol is sufficient to obtain well-detectable chromatographic peaks in all tested matrices. Furthermore, for quantification, the lower JA-*d_2_*-Ile peak area is closer to the peak areas of endogenous JA-Ile. The methodology was validated for accuracy and precision as described in [41].

Extraction and quantification of inactive and active endogenous BR were performed as described previously [43,44]. Samples (10 mg fresh weight) were extracted in ice-cold 60% acetonitrile, and 25 pmol deuterium-labeled internal standards of BRs (OlChemIm Ltd., Olomouc, Czech Republic) were added to each sample. After 12 h, samples were centrifuged (36,670 g, 15 min, 4 °C) and supernatants were purified using 50 mg Discovery DPA-6S cartridges (Supelco, Bellefonte, PA, USA). After evaporation to dryness, samples were reconstituted in 40 µL methanol and analyzed by liquid chromatography with tandem mass spectrometry (UHPLC-MS/MS) using an ACQUITY UPLC I-Class System (Waters, Milford, MA, USA) with a triple quadrupole mass spectrometer Xevo TQ-S MS (Waters MS Technologies, Manchester, UK).

### 2.5. Hydrogen Peroxide Analysis

Localization of H_2_O_2_ in leaf tissue was performed using 3,3-diaminobenzidine (DAB, Sigma-Aldrich, St. Louis, MO, USA) as described [45] and observed under a stereomicroscope (Zeiss SteREO Discovery.V20, Jena, Germany). The H_2_O_2_ content in leaves was determined spectrophotometrically using the TiOSO_4_ method [46]. Absorbance was measured at 405 nm.

### 2.6. Statistical Analysis

The number of biological replicates in each experiment is indicated in the figure captions. When comparing two mean values, a Student’s *t*-test was performed after checking the homogeneity of variance between the two samples with the F-test. Differences between the means of more than two groups were statistically assessed using two-way analysis of variance (ANOVA) followed by a post hoc Duncan’s multiple range test (DMRT).

## 3. Results

### 3.1. Differences in Transcriptomic Responses Between JA-Deficient, JA-Insensitive, and Wild-Type Plants Infected with PSTVd

**Figure 1:**
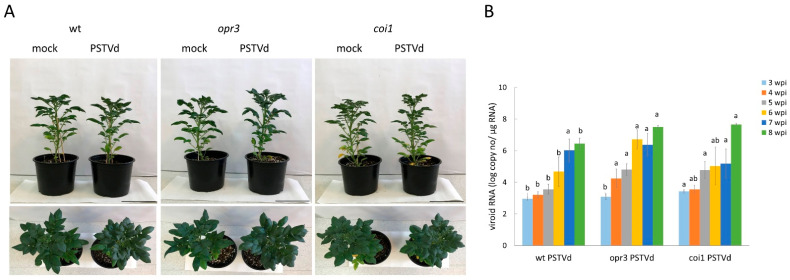
Symptom development and viroid RNA accumulation in *opr3*, *coi1*, and wild-type (wt) plants infected with PSTVd, compared to the corresponding mock-inoculated plants. (**A**) The first mild symptoms, such as stunted stem growth, epinasty, and pointed upper leaves, were observed in all three tested genotypes 5 weeks after inoculation (wpi); bar = 15 cm. (**B**) The dynamics of viroid RNA accumulation were determined by one-step RT-qPCR. At 8 wpi (experimental endpoint), the mean viroid load in *opr3* and *coi1* plants increased by 11-fold (3.15 × 10^7^ copies/μg RNA) and 15-fold (4.4 × 10^7^ copies/μg RNA), respectively, compared with wild-type plants (2.8 × 10^6^ copies/μg RNA). Values are means ± SE (*n* = 6) from one representative experiment. Different letters indicate significant differences among the three genotypes, assessed separately at each time point using DMRT (*p* < 0.05).

To identify genes and pathways specifically regulated by JA in response to PSTVd infection, transcriptomic profiles of PSTVd-infected leaves were compared with control leaves in wild-type, JA-deficient *opr3*, and JA-insensitive *coi1* lines. Samples were collected at 5 wpi, when symptoms began to appear (Figure 1A and Figure S1), and viroid load was significantly higher (*p* < 0.05) in *opr3* and *coi1* plants (6.3 × 10^5^ and 5.8 × 10^5^ copies/μg RNA, respectively), compared to wild-type plants (3.6 × 10^4^ copies/μg RNA) compared to wild-type plants (Figure 1B).

**Figure 2:**
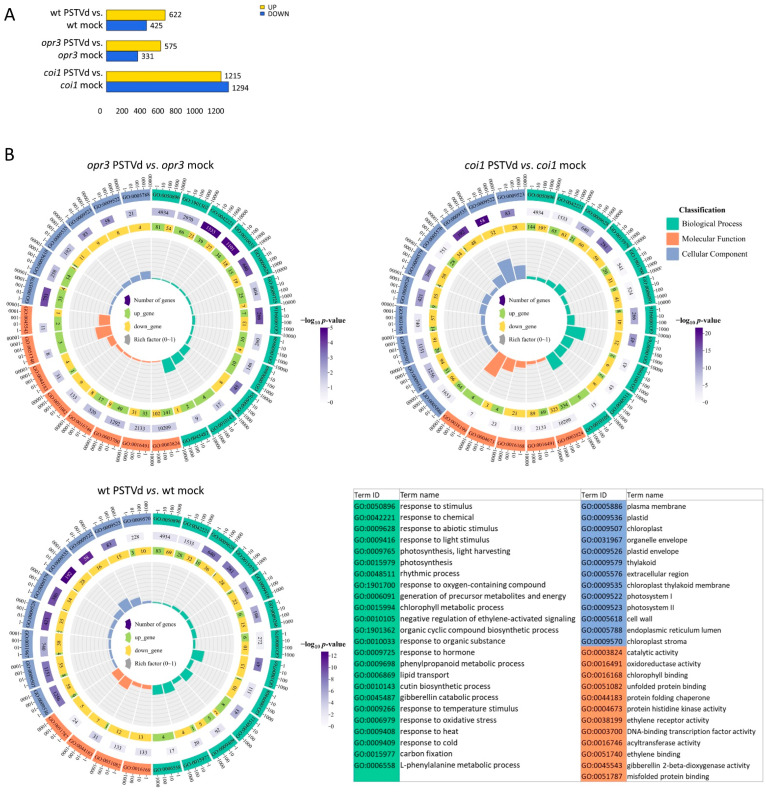
Transcriptional changes associated with PSTVd infection in *opr3*, *coi1*, and wild-type potato plants at 5 wpi. (**A**) The number of upregulated (UP) and downregulated (DOWN) DEGs (|log_2_fc| ≥ 2, *p* < 0.05) in three pairwise comparisons between PSTVd-inoculated and corresponding control (mock-inoculated) plants. (**B**) Top 26 enriched gene ontology (GO) terms for DEGs in the three comparisons; all data are in Supplementary Table S3. From the outermost to innermost circles, the first circle represents GO terms, with different colors indicating distinct terms. The second circle shows the total number of genes annotated to each term and the corresponding adjusted *p*-value, with deeper purple colors indicating smaller *p*-values. The third circle illustrates the ratio of upregulated to downregulated genes, with green representing the proportion of upregulated genes and yellow representing the proportion of downregulated genes; exact gene counts are labeled below. The fourth circle quantifies the enrichment factor, visualized in increments of 0.1, representing the ratio of DEGs to the total gene set within the given GO term.

Overall, twice as many DEGs were detected after PSTVd infection in *coi1* than in *opr3* or wild-type (2509, 906, and 1047 DEGs, respectively) (Figure 2). GO enrichment analysis revealed that the Biological Process (BP) GO terms related to responses to chemical and abiotic stimuli, and photosynthesis were significantly enriched in all examined lines after PSTVd infection (Figure 2, Table S3). However, the BP GO terms response to oxygen-containing compounds and negative regulation of ethylene signaling were enriched only in *coi1*, while response to hormone, phenylpropanoid metabolism, and lipid transport were enriched only in *opr3*. In the Molecular Function (MF) category, GO terms related to catalytic activity, oxidoreductase activity, and ethylene binding were enriched in *coi1* and *opr3*; chlorophyll binding was enriched in *coi1* and wild-type; while unfolded protein binding was enriched in *opr3* and wild-type. Among the Cellular Component (CC) GO terms, those linked to the chloroplast and thylakoid membrane were significantly enriched in all examined lines in response to PSTVd infection.

DEGs from the comparison between PSTVd- and mock-inoculated plants of all tested lines were assigned to MapMan functional classes. In the Biotic Stress overview, the most represented DEGs are related to signaling, proteolysis, and cell wall modification, followed by genes involved in transcriptional regulation, hormone signaling, and redox state (Figure S2, Table S4). Among DEGs encoding transcription factors (TFs), those for ethylene responsive factors (ERFs) and WRKYs were the most abundant in infected plants of all tested lines, as confirmed by PlantTFDB analysis (Figure S3). Additionally, activation of genes from the MYB and bHLH TF families was observed in wild-type and *opr3*, respectively. In *coi1*, a significant number of DEGs for ERF, MYB, and TCP TFs were downregulated compared to the wild-type after PSTVd infection. Besides stress responses and developmental processes, ERFs also participate in ET signaling. Regarding other hormone signaling pathways, more AUX-related genes were upregulated in *opr3* and *coi1* compared to the wild-type after PSTVd infection (Figure S2, Table S4).

### 3.2. Comparative Analysis of DEGs

**Figure 3:**
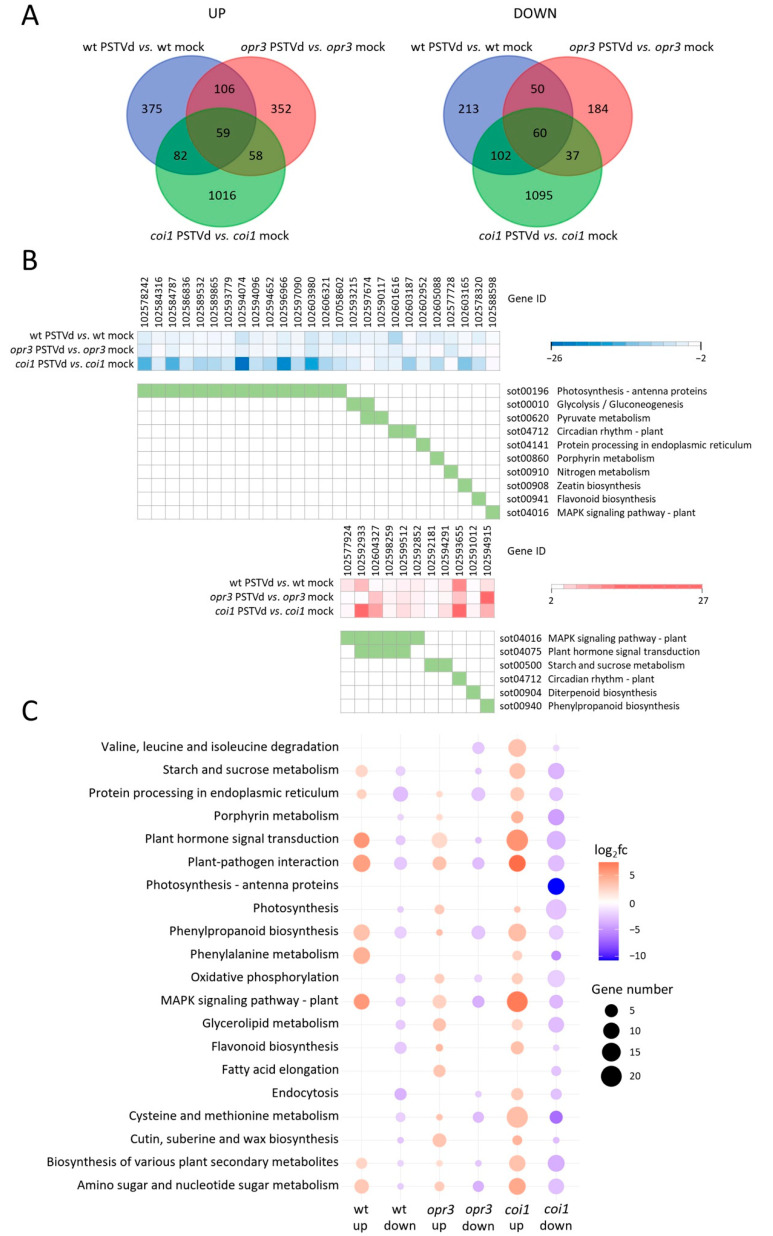
Overview of DEGs in PSTVd-infected *opr3*, *coi1*, and wild-type potato plants. (**A**) Venn diagrams show the overlap of DEGs upregulated or downregulated at 5 wpi in three comparisons between infected and control (mock-inoculated) plants. (**B**) Expression heatmaps and KEGG pathway analysis of DEGs shared among the three comparisons. (**C**) Top 20 KEGG pathways enriched with genotype-specific DEGs in the *opr3*, *coi1*, and wild-type comparisons; all data are in Supplementary Table S5.

The overlap between DEGs in PSTVd-infected and control samples of *opr3*, *coi1*, and wild-type plants is shown in Venn diagrams (Figure 3A). Functional KEGG analysis identified only 11 induced genes and 28 repressed genes common to both wild-type and transgenic lines, suggesting regulation by the same or overlapping pathways (Figure 3B, Table S5). Common upregulated genes were enriched in MAPK signaling and plant hormone signaling, including *MAPK7* and ET signaling genes (green ripe-like 1 *GRL1*, ethylene receptors *ETR2*-like and *EBF1*-like, and *ERF1B*-like). Common downregulated genes were enriched in photosynthesis-antenna proteins, glycolysis/gluconeogenesis, and pyruvate metabolism, indicating repression of primary metabolism after PSTVd infection.

Most genotype-specific DEGs detected after PSTVd infection in all comparisons were enriched in plant hormone signal transduction, plant-pathogen interaction, and MAPK signaling pathways, although the number of DEGs varied (Figure 3C). The *coi1*-specific DEGs related to hormone signaling were mainly involved in ET signaling (*ERF1B*-like), AUX signaling (Aux/IAAs, SAURs, and AUX-amido synthetase *GH3.6* and *GH3.1* genes), ABA signaling (ABA receptor *PYL4*-like, protein phosphatase 2C (*PP2C*)), GA signaling (*GID1B*-like), and SA signaling (regulatory protein *NPR3*-like and *TGA2*) (Table S5). The *opr3*-specific upregulated DEGs related to hormone signaling were mainly involved in AUX signaling (*GH3.5*, auxin transporter-like protein *LAX3*, and *AUX/IAA14*-like genes), JA signaling (*MYC4*-like), ET signaling (ETHYLENE INSENSITIVE 3-like 3 protein *EIL3* gene), and MAPK signaling (serine/threonine-protein kinase *SAPK3*-like), while genes related to CK biosynthesis and calcium signaling, were downregulated (Table S5). In addition, several more pathways were enriched with *coi1*-specific upregulated DEGs, such as cysteine and methionine metabolism (including ET biosynthesis genes *ACO* and *ACS*) and phenylpropanoid biosynthesis, while photosynthesis and oxidative phosphorylation pathways were enriched with downregulated *coi1*-specific DEGs. In contrast, only infected *opr3* plants showed upregulated DEGs assigned to glycerolipid metabolism, and cutin, suberin, and wax biosynthesis, while *opr3*-specific downregulated DEGs were assigned to protein processing and phenylpropanoid biosynthesis, including class III peroxidases (Figure 3C).

To gain better insight into the opposing regulation of defense responses in PSTVd-infected potato relative to JA-controlled pathways, we analyzed shared genes with contrasting expression between wild-type and transgenic lines (Figure S4A). KEGG analysis showed that DEGs upregulated in *coi1* but downregulated in wild-type were enriched in alanine, aspartate, and glutamate metabolism, as well as phenylpropanoid biosynthesis (Figure S4B, Table S6). In contrast, DEGs downregulated in *coi1* but upregulated in wild-type were enriched in pentose and glucuronate interconversions, amino sugar and nucleotide sugar metabolism, and plant-pathogen interaction pathways, highlighting the role of COI1-dependent JA signaling in the potato-PSTVd interaction. No enriched pathways were detected in the comparison between *opr3* and wild-type.

Identified DEGs were further analyzed using RT-qPCR. Transcript accumulation estimates for 15 selected genes from RNA-Seq closely matched those from RT-qPCR, indicating that the RNA-Seq data are reliable (Figure S5).

### 3.3. Expression Profiles of Hormone-Related, MAPK, and PR Genes

**Figure 4:**
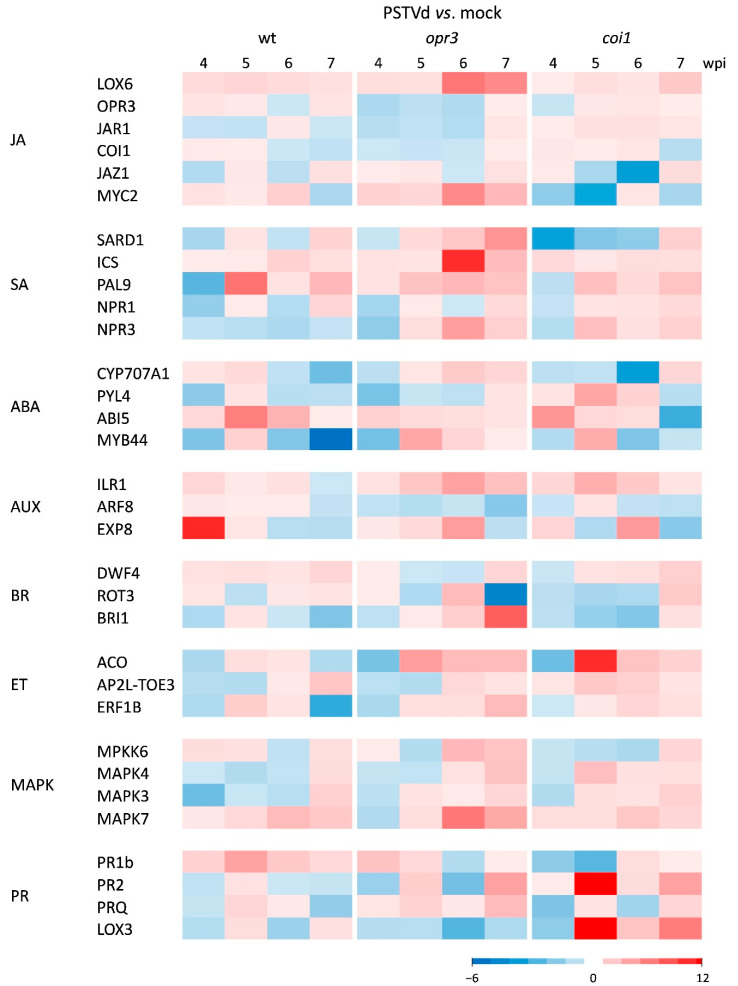
Temporal dynamics of hormone- and defense-related gene expression in leaves of *opr3*, *coi1*, and wild-type (wt) plants in response to PSTVd infection. Heatmaps display changes in relative gene expression at 4, 5, 6, and 7 wpi, as determined by RT-qPCR. The color scale on the heatmap indicates the mean log_2_fc from three biological replicates, normalized to the expression of the reference gene *EFα1*, and calculated relative to the corresponding control (mock-inoculated plants). Statistical evaluation of the data is in Table S7. Details on the genes and primers are in Table S1.

**Figure 5:**
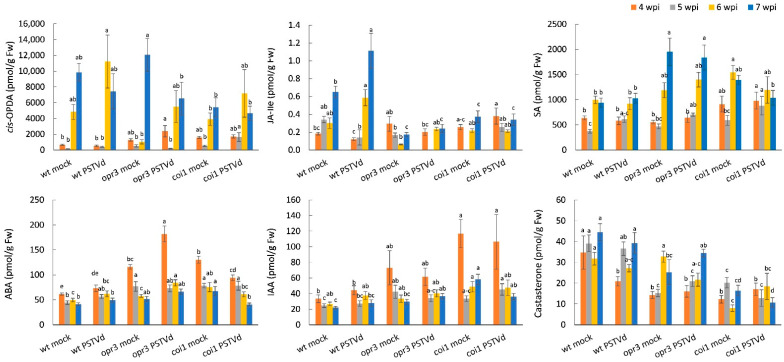
Hormone accumulation in leaves of *opr3*, *coi1*, and wild-type plants during PSTVd infection. Concentrations (pmol/g Fw) of *cis*-(+)-12-oxo-phytodienoic acid (*cis*-OPDA), jasmonyl-isoleucine (JA-Ile), salicylic acid (SA), abscisic acid (ABA), indole-3-acetic acid (IAA), and castasterone (CS) were quantified at different time points. Values are means ± SE (*n* = 6–8) pooled from two independent experiments. Different letters indicate significant differences among six plant groups (three genotypes; both PSTVd- and mock-inoculated), assessed at each time point separately using DMRT (*p* < 0.05).

According to KEGG functional analysis, most genotype-specific DEGs in all comparisons were enriched in plant hormone signal transduction, plant-pathogen interaction, and MAPK signaling pathways. These differences were validated with independent data from RT-qPCR and hormone analyses. The dynamic expression patterns of selected phytohormone-related genes were analyzed at different time points, before and after symptom onset (Figure 4, Table S7). The same tissue samples were used for hormone quantification (Figure 5).

After PSTVd infection, earlier activation of JA biosynthesis and perception genes (*LOX6*, *OPR3*, *JAR1*, and *COI1*) was detected in wild-type and *coi1* plants compared to *opr3* plants. Conversely, activation of JA signaling genes (*JAZ1* and *MYC2*) was stronger in infected *opr3* plants. Hormone analysis showed that the level of *cis*-OPDA, a precursor of JA-Ile, peaked at 6 wpi and then decreased at 7 wpi in all tested lines, while significant JA-Ile accumulation was detected only in infected wild-type plants. SA biosynthesis (*ICS* and *PAL9*) and SA signaling (*SARD1* and *NPR1*) genes were upregulated in all tested lines (*opr3* > *coi1* > wt); however, SA levels remained unchanged. ABA content and ABA hydroxylase gene expression increased only in *opr3* plants at the early stage of infection, while increased expression of *ABI5* and *MYB44* suggested activation of ABA signaling in all tested lines. The relative expression of auxin-responsive genes (*ILR1*, *ARF8*, and *EXP8*) was stronger in *opr3* and *coi1* compared to wild-type, while IAA content increased slightly in *opr3* and wild-type but decreased in *coi1*, after PSTVd infection. PSTVd-induced activation of the BR receptor gene *BRI1* was higher in *opr3*, coinciding with a slight increase in castasterone (CS) content. The relative expression of ET biosynthesis (*ACO*) and ET signaling (*ERF1B* and *AP2-TOE3*) genes was elevated in all tested lines at 5–7 wpi (*coi1* > *opr3* > wt).

Since cross-talk among MAPK, hormone, and ROS signaling pathways is important for the fine-tuned modulation of plant immunity [47,48], we measured the time course of expression of selected *MAPK* and *PR* protein genes after PSTVd infection (Figure 4, Table S7). Results showed activation of *MPKK6*, *MAPK4*, and *MAPK3* genes in wild-type plants at the early stage, but in *opr3* and *coi1* plants at the late stage of infection. Expression of the gene for MAPK7, an H_2_O_2_-responsive MAPK [49], was upregulated in all tested lines. Relative *PR1b* gene expression was stronger in wild-type plants, while *PR2* and *PRQ* gene expression was stronger in *opr3* and *coi1* plants, after PSTVd infection. Activation of *LOX3*, a pathogen-responsive lipoxygenase, was strongest in infected *coi1* plants.

### 3.4. Regulation of Redox Processes in opr3, coi1, and Wild-Type Potato Plants During PSTVd Infection

**Figure 6:**
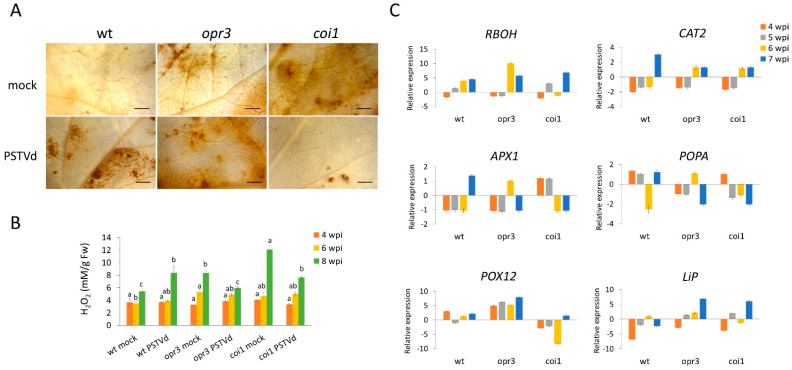
Dynamics of antioxidative responses in *opr3*, *coi1*, and wild-type plants during PSTVd infection. (**A**) H_2_O_2_ accumulation in leaves of PSTVd- and mock-inoculated plants at 8 wpi was localized by DAB staining, and observed under a stereomicroscope; bar = 2 mm. (**B**) H_2_O_2_ accumulation (mM/g Fw) at different time points was quantified spectrophotometrically using the TiOSO_4_ method. Values are means ± SE (*n* = 4) from one representative experiment. Different letters indicate significant differences among six plant groups (three genotypes; both PSTVd- and mock-inoculated), assessed at each time point separately using DMRT (*p* < 0.05). (**C**) The expression pattern of H_2_O_2_-producing and scavenging genes was analyzed by RT-qPCR. Values represent the mean log_2_fc ± SE (*n* = 3). Statistical evaluation of the data is in Table S7.

To investigate the mechanisms underlying altered H_2_O_2_ accumulation in JA-deficient plants, we analyzed transcript levels of ROS-producing (*RBOH*) and ROS-scavenging genes, including catalase (*CAT2*), ascorbate peroxidase (*APX1*), suberization-associated anionic peroxidase (*POPA*), lignin-forming anionic peroxidase (*LiP*), and peroxidase 12 (*POX12*). After PSTVd infection, wild-type plants accumulated H_2_O_2_, while *opr3* and *coi1* plants showed reduced H_2_O_2_ levels at the late stage of infection (Figure 6A,B). RT-qPCR revealed earlier activation of *RBOH*, *CAT2*, and *APX1* genes in *coi1* and *opr3* plants compared to wild-type plants (Figure 6C, Table S7). For Class III peroxidases, a plant-specific family of antioxidant enzymes involved in diverse functions [50], *POPA* expression was upregulated in wild-type plants, *LiP* was upregulated in *coi1* and *opr3* plants, and *POX12* expression was upregulated in *opr3* plants, but downregulated in *coi1* plants.

### 3.5. Effect of MeJA Treatment on Defense Responses in PSTVd-Infected Potato

**Figure 7:**
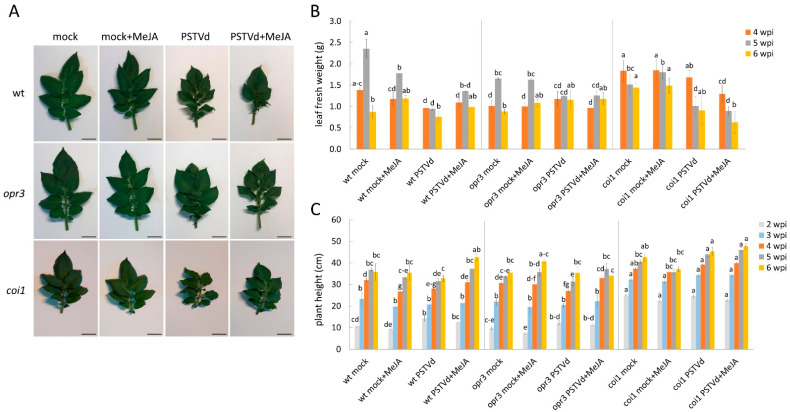
Effects of MeJA pretreatment on symptom appearance in *opr3*, *coi1*, and wild-type plants infected with PSTVd. (**A**) Morphology of upper potato leaves in PSTVd- or mock-inoculated plants in response to MeJA, examined at 6 wpi; bar = 1 cm. (**B**) Growth (g Fw) of upper systemically infected leaves was examined at different time points after MeJA treatment, with or without PSTVd infection. (**C**) Stem elongation was determined at different time points. Values are means ± SE (*n* = 4–6) from one representative experiment. Different letters indicate significant differences among 12 plant groups (three plant lines; both mock and infected, with or without MeJA), assessed at each time point separately using DMRT (*p* < 0.05). Untreated mock plants (mock); MeJA-treated mock plants (mock + MeJA); PSTVd-infected untreated (PSTVd); MeJA-treated PSTVd-infected (PSTVd + MeJA).

Treatment with MeJA alleviated the negative effects of PSTVd infection on apical leaf and stem growth in wild-type plants, but had no significant effect on symptom appearance in *opr3* and *coi1* plants (Figure 7 and Figure S6).

**Figure 8:**
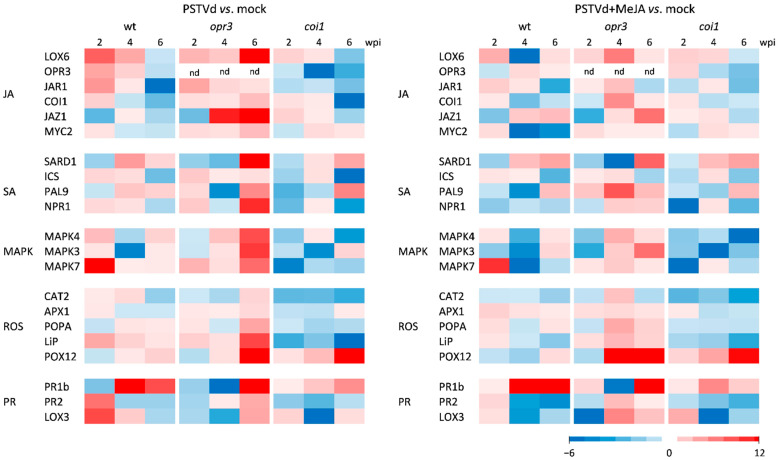
Effects of MeJA pretreatment on hormone- and defense-related gene expression in *opr3*, *coi1*, and wild-type (wt) plants infected with PSTVd. Relative gene expression in leaves was analyzed at different time points by RT-qPCR. The color scale on heatmaps indicates the mean log_2_fc from three biological replicates, normalized to the expression of the reference gene *EFα1*, and calculated relative to the corresponding control (mock plants pretreated with H_2_O). Statistical evaluation of the data is in Table S8.

In PSTVd-infected wild-type plants, MeJA caused a transient decrease in the expression of nearly all tested genes involved in JA, SA, MAPK, and ROS signaling (Figure 8, Table S8), along with an increase in H_2_O_2_ content (Figure S6). In PSTVd-infected *opr3* plants, MeJA initially decreased but then increased the expression of JA-related and SA-related genes, compared to untreated infected *opr3* plants. A decrease in H_2_O_2_ content was also observed. As expected, MeJA had little effect on the expression of selected genes and H_2_O_2_ content in JA-insensitive *coi1* plants. Despite its notable impact on gene expression and symptom development, MeJA did not significantly affect the dynamics of PSTVd RNA accumulation in any of the tested lines (Figure S6).

## 4. Discussion

Functional analyses of differentially expressed genes (DEGs) showed that PSTVd infection in all tested potato lines suppressed genes related to photosynthesis and primary metabolism, while activating genes involved in proteolysis, signaling, and defense responses. This indicates a redirection of energy from metabolic processes to defense, a widespread and complex feature of pathogen-infected plants [23,51,52]. The late stage of infection in wild-type plants was characterized by the accumulation of JA-Ile, consistent with our previous results [25]. As expected, the transgenic *opr3* and *coi1* plants produced less JA-Ile than the wild-type, but no significant increase in JA-Ile levels in response to PSTVd was observed. However, the relative expression of the JA signaling gene *MYC2* was higher in *opr3* plants, while the expression of early JA biosynthesis genes such as *LOX* was higher in *coi1* plants. We hypothesize that this may be related to increased production of other jasmonates and oxylipins [10,14]. Both *opr3* and *coi1* transgenic lines had higher viroid loads than the wild-type, suggesting that JA is important for the basal defense response of potato against PSTVd. Transient accumulation of *cis*-OPDA, a precursor of JA-Ile and a JA-independent signaling molecule [53], was observed in infected plants of all tested lines. Therefore, it likely did not contribute to the difference in susceptibility to PSTVd among the tested lines, which requires further investigation.

Consistent with its defensive role, JA accumulation may inhibit plant growth [54]. When infected with PSTVd, JA-deficient *opr3* plants showed milder symptoms in leaf and stem growth, while JA-insensitive *coi1* plants exhibited significantly elongated stems, contrary to the typical PSTVd symptom of stunted stem growth. Detailed analysis of PSTVd-induced transcriptomic, hormone, and physiological responses in transgenic *opr3* and *coi1* lines, compared to the wild-type line, revealed several phenomena possibly associated with altered basal defense responses and symptom development in these lines: (i) activation of other hormone signaling pathways and their interactions with the JA signaling pathway; (ii) alteration of MAPK signaling, transcription factors, and *PR* gene expression; and (iii) transcriptional modification of antioxidant responses and metabolic pathways.

### 4.1. Activation of Other Hormone Signaling Pathways and Their Interactions with the JA Signaling Pathway

An imbalance between JA and AUX pathways can increase plant susceptibility to pathogens because high AUX levels promote cell growth, facilitating pathogen invasion and reducing the plant’s ability to activate JA-mediated defense responses [55]. At the transcriptome level, PSTVd-infected *opr3* plants showed increased expression of several AUX metabolism and early AUX signaling genes, including *LAX3*, *SAUR*s, and *AUX/IAA* genes [56]. For example, expression of *StGH3.1* (an ortholog of *AtGH3.1* and *OsGH3.8* [57] was upregulated in JA-insensitive and JA-deficient plants compared to wild-type plants, suggesting that JA negatively regulates *GH3.1* expression in response to PSTVd in potato. An interplay between JA and AUX signaling pathways during potato-PSTVd interaction is also suggested by the differential expression of auxin-responsive genes *ARF8* [56] and *EXP8* [58] in *opr3* and *coi1* lines compared to wild-type plants. We hypothesize that impaired JA biosynthesis and signaling affect the regulation of AUX signaling, contributing to symptom alleviation in *opr3* and *coi1* plants to PSTVd infection. However, auxin content remains relatively unchanged after PSTVd infection in all lines tested.

Abscisic acid mediates abiotic stress tolerance and regulates growth and development, but its role in disease resistance is complex and depends on the specific plant-pathogen interaction [5]. Jasmonate deficiency increases stress sensitivity in rice, and ABA biosynthesis or catabolism in response to osmotic stress can be modulated by JA [59]. Similarly, ABA accumulation in the early stage of PSTVd infection and increased expression of genes encoding the transcription factors MYB44 and MYC2 suggest enhanced stress response and stomatal regulation in JA-deficient *opr3* plants. Both MYB44 and MYC2 contribute to stomatal closure under stress conditions; however, MYC2 acts through JA signaling and stimulation of ABA biosynthesis [60], while MYB44 acts through ABA signaling [61]. Multifunctional MYB44 can also act as a negative regulator of ABA signaling in leaf senescence [61]. In contrast, in JA-insensitive *coi1* plants, ABA levels decreased, while the expression of genes for ABA receptors PYR1 and PYL4 [62] and the transcription factor ABI5-like protein 5 increased, suggesting activation of ABA signaling in response to PSTVd infection. ABA receptors and ABI transcription factors can, in turn, influence other signaling pathways, including the JA pathway, to fine-tune downstream stress responses and growth regulation [63].

Ethylene can have both negative and positive effects on plant defense responses and often acts synergistically with JA to activate defense-related genes [6,64]. Coordinated crosstalk between ET and JA converges at the transcriptional activation of *ERF1*. Arabidopsis *ERF1* is an early JA-responsive gene in the ERF branch of the JA defense response pathway, while ethylene promotes a longer-lasting induction of *ERF1* expression [64,65]. The AP2/ERFs, in addition to their role in hormone crosstalk, are also recognized for mediating plant responses to biotic stress, as transcriptional activators or repressors [66,67]. About half of the PSTVd-induced genes shared by wild-type, *opr3*, and *coi1* lines encode ET-responsive proteins (GRL1, ETR2-like, EBF1-like, and ERF1B-like), suggesting that activation of ET signaling is a core response to PSTVd in potato. However, the total number of ET-related DEGs in response to PSTVd was significantly higher in *opr3* and especially in *coi1* plants, which may also suggest an alternative, JA-independent activation of ET signaling and/or that *COI1* is not completely silenced [26]. The involvement of ET signaling in other plant-viroid interactions has also been reported [11,17,68,69,70], while ET accumulation was mostly associated with symptom development [71]. To clarify the role of ET in the potato response to PSTVd, future studies should analyze ET content.

Several lines of evidence indicate that suppression of GA biosynthesis genes, which is associated with reduced GA responses, plays a major role in the development of stunting in viroid-infected plants [16,17,24,56,68,72]. Our study showed downregulation of the GA receptor *GID1B* gene, and upregulation of GA-responsive *RSI-1* genes after PSTVd infection in wild-type and *opr3* plants, while in *coi1* plants, the infection was accompanied by opposite responses. *StRSI-1* is an ortholog of *AtGASA5* (Gibberellin-stimulated transcript GAST1 protein homolog 5), a downstream gene of *DELLA*, which acts as a suppressor of gibberellin responses and stem growth in *Arabidopsis thaliana* [73]. In addition, GASA proteins are involved in JA-dependent biotic defense responses [74,75]. Therefore, we hypothesize that the increased expression of *RSI-1* in PSTVd-infected *coi1* plants may be associated with the absence of stem stunting symptoms in *coi1* plants, as part of the crosstalk between growth-promoting GA signaling and defense-promoting JA signaling [54].

Regarding the other hormones analyzed in this study, transcriptomic data showed a limited effect of PSTVd infection on the activation of SA and BR biosynthesis genes. This is consistent with the small changes in endogenous SA and CS content measured in infected compared to uninfected plants across all three lines. However, RT-qPCR analysis detected increased expression of SA biosynthesis (*ICS* and *PAL9*) and SA signaling (*NPR1* and *NPR3*) genes in wild-type plants during the early stage, and in *opr3* and *coi1* plants during the late stage of PSTVd infection. Unlike *Arabidopsis*, in some plant species such as potato and rice, SA levels do not increase significantly after pathogen infection, but the SA signaling pathway remains active and contributes to disease resistance [76,77].

Interestingly, transcriptome analysis also revealed contrasting expression of genes involved in the biosynthesis and metabolism of growth-promoting hormones CK (*IPT*- and *ZOG*-like) and PSK (*phytosulfokines 3-like*) in *opr3* and *coi1* plants infected with PSTVd. This suggests a possible interaction between CK, PSK, and JA signaling [78] during potato-PSTVd interaction, but further investigation is required.

### 4.2. Alteration of MAPK Signaling, Transcription Factors, and PR Genes in Response to PSTVd

MAPK cascades have been reported to regulate JA biosynthesis and JA-dependent gene expression [79]. JA also regulates both MAPK activity and *MAPK* gene expression, and silencing JA-related genes can affect MAPK activity [48]. After PSTVd infection, late activation of *MAPK3* and *MAPK4* genes was observed in *opr3* and *coi1* plants compared to wild-type plants, while pretreatment with MeJA accelerated gene activation in *opr3* plants. In *Arabidopsis*, *MAPK4* acts as a negative regulator of SAR and SA signaling but as a positive regulator of JA-dependent gene expression [80]. Our transcriptome analysis also revealed *coi1*-specific activation of a *MAP3KA*-like gene [81] and *opr3*-specific activation of *SAPK3*-like and *MAP2K*-like genes [82], suggesting their pathogen-dependent but JA-independent activation in potato. In addition, we detected PSTVd-induced expression of *MAPK7* in all tested potato lines, in all tested lines, which may be related to changes in JA and H_2_O_2_ status [49] associated with PSTVd infection.

By integrating signals from both hormones and ROS, the MAPK pathways can orchestrate a more precise and efficient plant defense response [47,83]. MapMan analysis showed that most defense-related genes, except miraculin [84], were upregulated in wild-type and opr3 plants compared to coi1 plants after PSTVd infection. Additionally, the expression of *PR1* genes [85] was lower and *PR2* genes [86] higher in *opr3* and *coi1* plants, which may be related to their higher susceptibility to PSTVd compared to wild-type plants, as indicated by viroid RNA accumulation. These results suggest the activation of alternative defense responses in *opr3* and *coi1* plants and highlight the role of JA signaling in regulating *PR* gene expression during PSTVd infection, possibly through interactions with other signaling pathways, including the ET and SA pathways [87].

Transcription factors (TFs) play a key role in establishing plant defense and symptom development during viroid infections [56,72]. The importance of bHLH TFs, including MYCs that directly interact with JA-responsive genes, in the tomato-PSTVd interaction has also been reported [88]. In our study, the primary TF families showing significant responses to PSTVd in potato were identified, and the potential role of certain TFs in regulating hormone, MAPK, and ROS signaling during the potato-PSTVd interaction is discussed. However, given the numerous TFs and their diverse roles in regulating various defense-related genes, further research on specific TFs in the potato-PSTVd interaction is needed.

### 4.3. Transcriptional Modification of Antioxidant Responses and Metabolic Pathways in opr3 and coi1 Plants During PSTVd Infection

The physiological effects of JA include activation of ROS signaling pathways and recruitment of the cellular antioxidant defense system to maintain moderate ROS levels [89]. After PSTVd infection, DEGs for enzymes that regulate the cellular redox state, including APX [90], were mainly downregulated in *opr3* and *coi1* compared to wild-type. Since *opr3* and *coi1* plants accumulate less H_2_O_2_ than wild-type plants after PSTVd infection, their reliance on APX is less pronounced. However, not all antioxidant responses followed this pattern. DEGs for glutathione S-transferases were mainly induced by PSTVd infection in *opr3* and especially in *coi1*, suggesting their involvement in ROS reduction in plants with compromised JA pathways. JA signaling mediated by COI1 is important for controlling ROS accumulation by influencing the activity of MYC proteins, which regulate the expression of genes encoding antioxidant enzymes, including NOX/RBOH and POX12, involved in ROS production during plant defense against pathogens [89,91]. In PSTVd-infected *opr3* plants, one of the most prominent features was increased expression of *MYC2*, *RBOH*, and *POX12* genes, whereas in infected *coi1* plants, both *MYC2* and *POX12* expression were reduced. Treatment with MeJA followed by PSTVd infection induced short-term H_2_O_2_ production, enhancing *POX12* expression, which in turn reduced H_2_O_2_ content in *opr3* plants at the late infection stage. High concentrations of MeJA can act pro-oxidatively to stimulate activation of the antioxidant system and some components of basal defense responses [92,93]. Reduced H_2_O_2_ accumulation may enhance the plant’s tolerance to the initial stressor and reduce symptom development, but may also promote pathogen progression [94].

Among other PSTVd-induced *POX* genes, *LiP* and *POPA* encode enzymes that use H_2_O_2_ as a substrate for lignification and suberinization, respectively. *LiP* and *POPA* showed contrasting expression patterns in wild-type and *opr3* compared with *coi1* plants. Increased expression of *LiP*, along with the lignin biosynthesis genes *4CL* and *CCoAOMT*, suggests increased H_2_O_2_ consumption during monolignol biosynthesis in infected *coi1* plants. Lignin is a complex polyphenolic polymer that serves as a structural component in plant cell walls, providing rigidity and strength, and acts as a physical and chemical barrier in basal plant immunity [95]. Lignification is negatively regulated by JA [92], which is consistent with the observed increased expression of lignification-related genes in *coi1* plants. In contrast, several *PAL* genes were specifically upregulated in wild-type plants after PSTVd infection, suggesting the production of various other phenolic secondary metabolites [96]. Altered expression of many genes encoding lignin-forming peroxidases and genes from the lignin-specific pathway was also observed in PSTVd-infected tomato [18].

Plants with impaired JA biosynthesis or signaling often activate alternative metabolic pathways, due to a bypass in the JA biosynthesis pathway, allowing accumulation of alternative defense compounds [7]. In PSTVd-infected *opr3* plants, genotype-specific activation of genes related to cutin, suberin, and wax biosynthesis (*CYP94A1* and feruloyl transferase *FHT*), and glycerophospholipid metabolism (glycerol-3-phosphate acyltransferases GPATs), was observed. Glycerolipids are important components of plant membranes, which are essential for development, growth, photosynthesis, and stress responses [97]. Cutin and suberin are protective polyester barriers formed by the polymerization of oxygenated fatty acids and glycerol [98]. Because glycerolipids and JA share fatty acids as precursors, inhibition of one pathway enhances regulation of the other, strengthening alternative defense pathways [99,100]. Therefore, we hypothesize that silencing *OPR3* alters glycerolipid and oxylipin metabolism and promotes cutin, wax, and suberin production in potato in response to PSTVd and increased osmotic stress in JA-deficient plants [59].

## 5. Conclusions

This study showed that silencing JA biosynthesis or JA signaling significantly alters the reprogramming of multiple signaling pathways, including hormone, MAPK, and ROS signaling, which disrupts defense regulation and increases susceptibility to PSTVd in potato. JA deficiency at the late stage of infection reduced symptom development in *opr3* plants, while MeJA treatment at the early stage of infection reduced symptoms in wild-type plants. The weak *coi1* response also supports COI1-dependent MeJA action. However, because only one MeJA dose and timing were used, conclusions about timing and dose–response are limited. Further studies, such as testing different MeJA concentrations, applying stage-specific inhibitory treatments, and conducting more detailed time-course sampling, should be conducted to provide more direct evidence supporting a positive role for JA in the early stage of PSTVd infection. Numerous transcriptional changes caused by PSTVd infection in *coi1* plants indicate the activation of alternative responses that compensate for compromised JA signaling, leading to symptom relief but increased viroid accumulation. This is consistent with the established role of JA as a regulator in the trade-off between growth and defense in plants [101]. Overall, these results enhance our understanding of the role of JA in basal immune responses against PSTVd and provide valuable gene resources for future functional analyses of key genes and regulatory mechanisms underlying immune responses to this pathogen in potato.

## Data Availability

The RNA-Seq data have been deposited in the NCBI database, with SRA accession code PRJNA1137360. Additional data supporting the conclusions of this article are included in the article and its Supplementary Files. Further inquiries can be directed to the corresponding author.

## Supplementary Materials

The following supporting information can be downloaded at: https://www.mdpi.com/article/10.3390/antiox15010086/s1, Figure S1: Effects of PSTVd infection on potato stem and leaf growth in *opr3* and *coi1* plants compared to wild-type plants; Figure S2: MapMan visualization of DEGs related to biotic stress in *opr3*, *coi1*, and wild-type potato plants during PSTVd infection; Figure S3: Distribution of DEGs in 10 major transcription factor families; Figure S4: Analysis of DEGs with contrasting regulation between opr3, coi1, and wild-type potato plants infected with PSTVd; Figure S5: RT-qPCR validation of RNA-Seq results; Figure S6: Effects of MeJA pretreatment on symptom development, H_2_O_2_ accumulation, and viroid RNA accumulation in *opr3*, *coi1*, and wild-type plants infected with PSTVd; Table S1: List of primers used in this study; Table S2: Raw and mapped statistics of RNA-Seq data; Table S3: GO enrichment analysis of DEGs; Table S4: MapMan analysis of DEGs associated with biotic stress, in PSTVd-infected versus control samples of wild-type, *opr3*, and *coi1* plants; Table S5: KEGG classification of genotype-specific and common DEGs; Table S6: KEGG classification of contrasting DEGs between different comparisons; Table S7: Expression profiles of genes involved in hormone- and defense-related responses determined by RT-qPCR; Table S8: Expression of hormone-, MAPK-, and ROS-related genes, in response to MeJA treatment and PSTVd infection.
