# Supplementary material for: Transcriptome and Hormone Analysis Revealed Jasmonic Acid-Mediated Immune Responses of Potato (Solanum tuberosum) to Potato Spindle Tuber Viroid Infection
Source: Antioxidants (Basel). 2026 Jan 8;15(1):86. doi: 10.3390/antiox15010086 (PMC12838034; doi:10.3390/antiox15010086)
Supplement: Supplementary file 1 [file antioxidants-15-00086-s001.zip › antioxidants-4000905-supplementary/Supplementary Figures S1-S6 (Markovic et al) R2.pdf]

SUPPLEMENTARY FIGURES

for

Transcriptome and hormone analysis revealed jasmonic acid-mediated immune responses of potato (*Solanum tuberosum*) to potato spindle tuber viroid infection

Iva Marković[1†], Bernard Jarić[1†], Jana Oklešťková [2], Jitka Široká[2], Kristina Majsec[1], Jasna Milanović[3], Snježana Kereša[4], Ivanka Habuš Jerčić[4], Ondřej Novák[2], Snježana Mihaljević[1*]

[1] Division of Molecular Biology, Ruđer Bošković Institute, Bijenicka cesta 54, 10000 Zagreb, Croatia

[2] Laboratory of Growth Regulators, Faculty of Science, Palacký University & Institute of Experimental Botany, Czech Academy of Sciences, Šlechtitelů 27,77900, Olomouc, Czech Republic

[3] Centre for Plant Protection, Croatian Agency for Agriculture and Food, Gorice 68b, 10000 Zagreb, Croatia

[4] Division of Plant Science, Faculty of Agriculture, University of Zagreb, Svetošimunska cesta 25, HR-10000 Zagreb

[†] These authors contributed equally to this work.

* For correspondence (snjezana.mihaljevic@irb.hr)

This .doc file includes Figures S1 to S6.

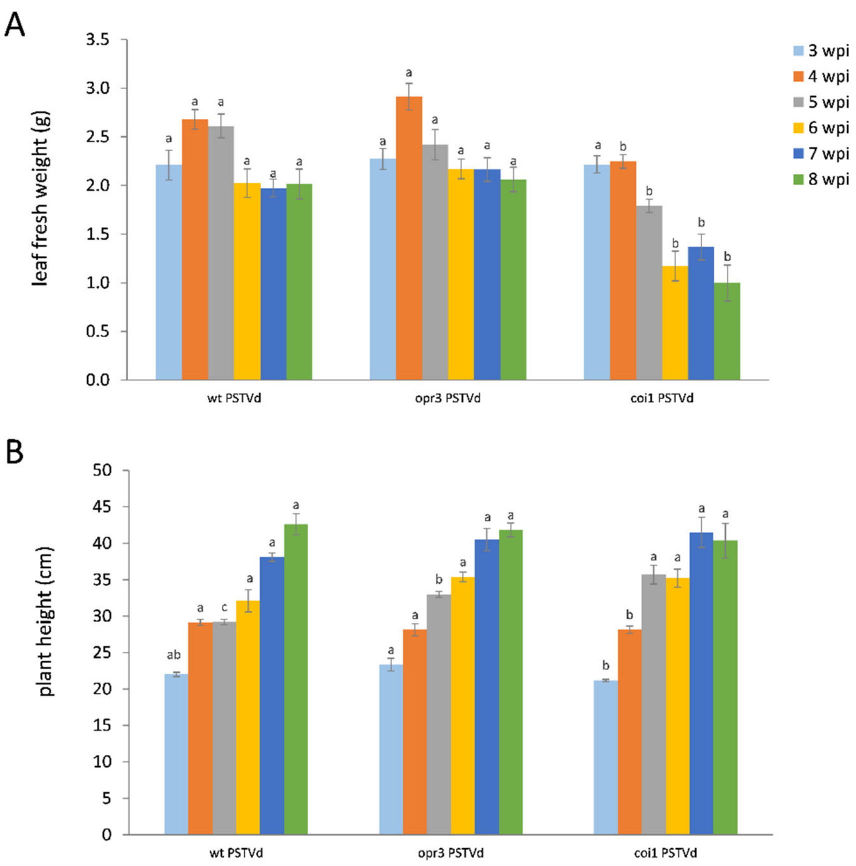

Figure S1. Effects of PSTVd infection on potato stem and leaf growth in *opr3* and *coi1* transgenic plants compared to wild-type plants. (**A**) Growth of upper young leaves at different time points after inoculation. (**B**) Comparison of plant height at different time points after inoculation; values are means ± SE (n = 6). Different letters indicate significant differences among the three genotypes, assessed separately at each time point using DMRT ($p < 0.05$).

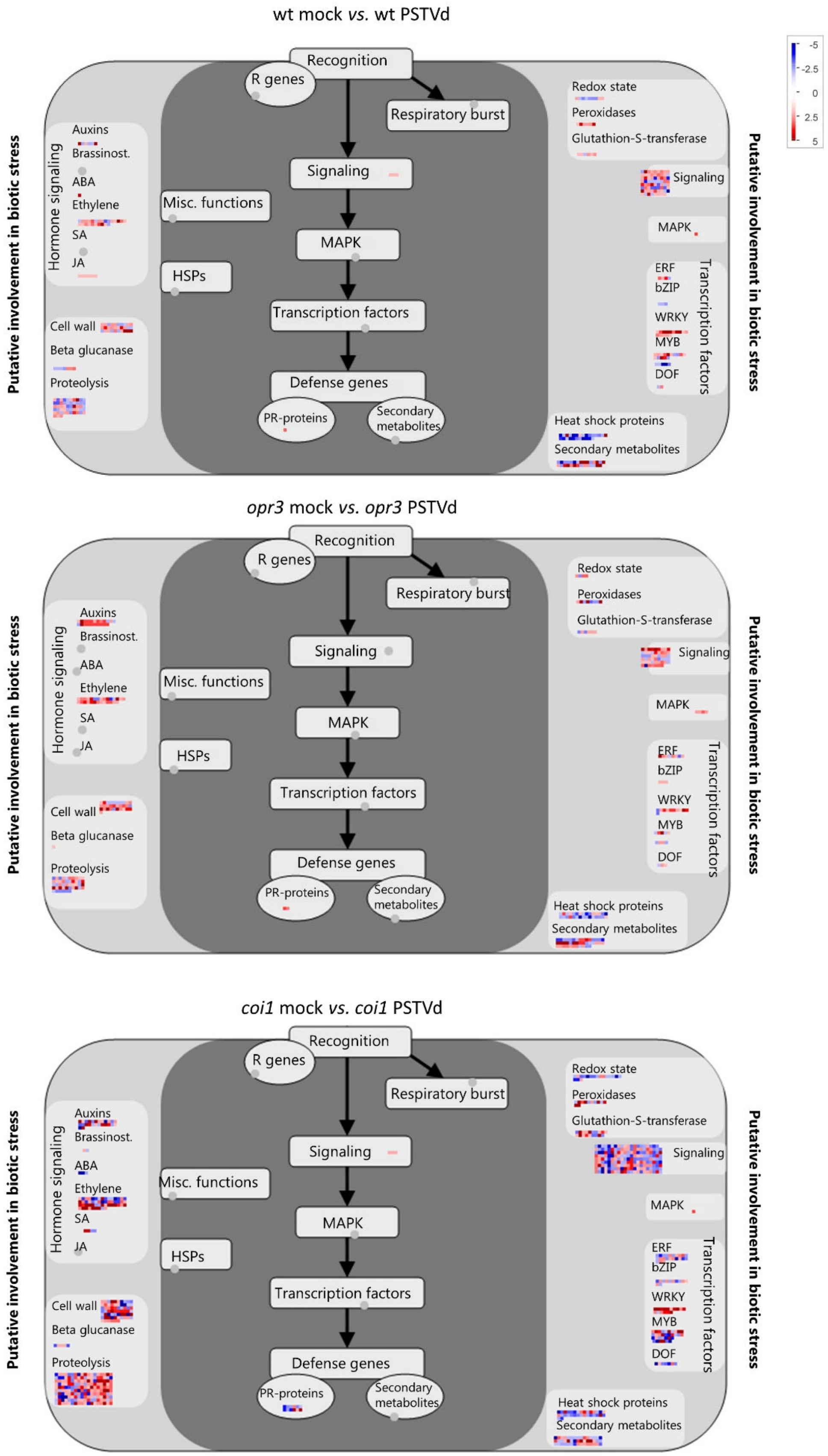

Figure S2. MapMan visualization of DEGs related to biotic stress in *opr3*, *coi1*, and wild-type potato plants during PSTVd infection. Red and blue squares represent DEGs ($|log_2fc| \geq 2$) that were upregulated and downregulated, respectively, in PSTVd-inoculated plants compared to the corresponding control (mock-inoculated) plants at 5 wpi. The color scale is shown as log_2fc from -5 to 5.

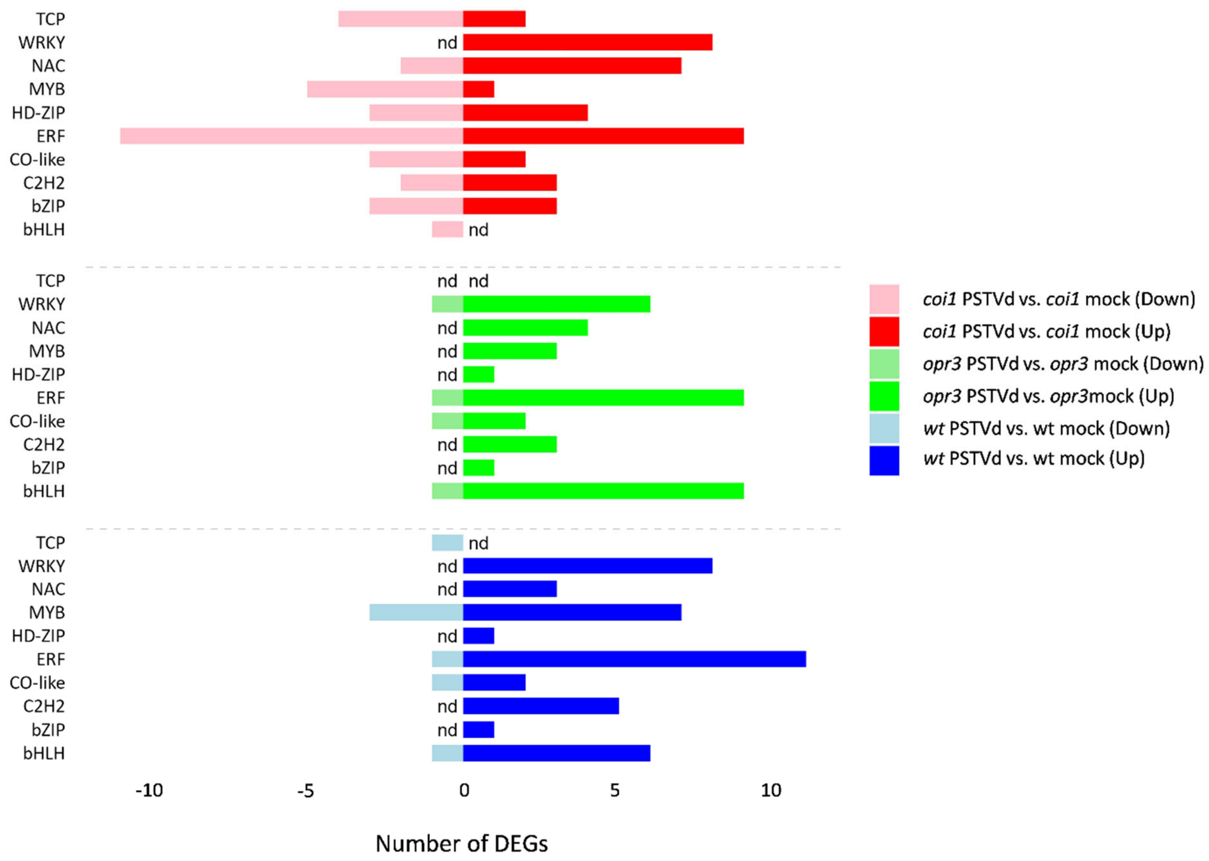

Figure S3. Distribution of DEGs in 10 major transcription factor families. DEGs were identified in PSTVd-infected *opr3*, *coi1*, and wild-type potato plants at 5 wpi compared to their respective controls. Negative values on the X-axis indicate the number of genes with downregulated expression.

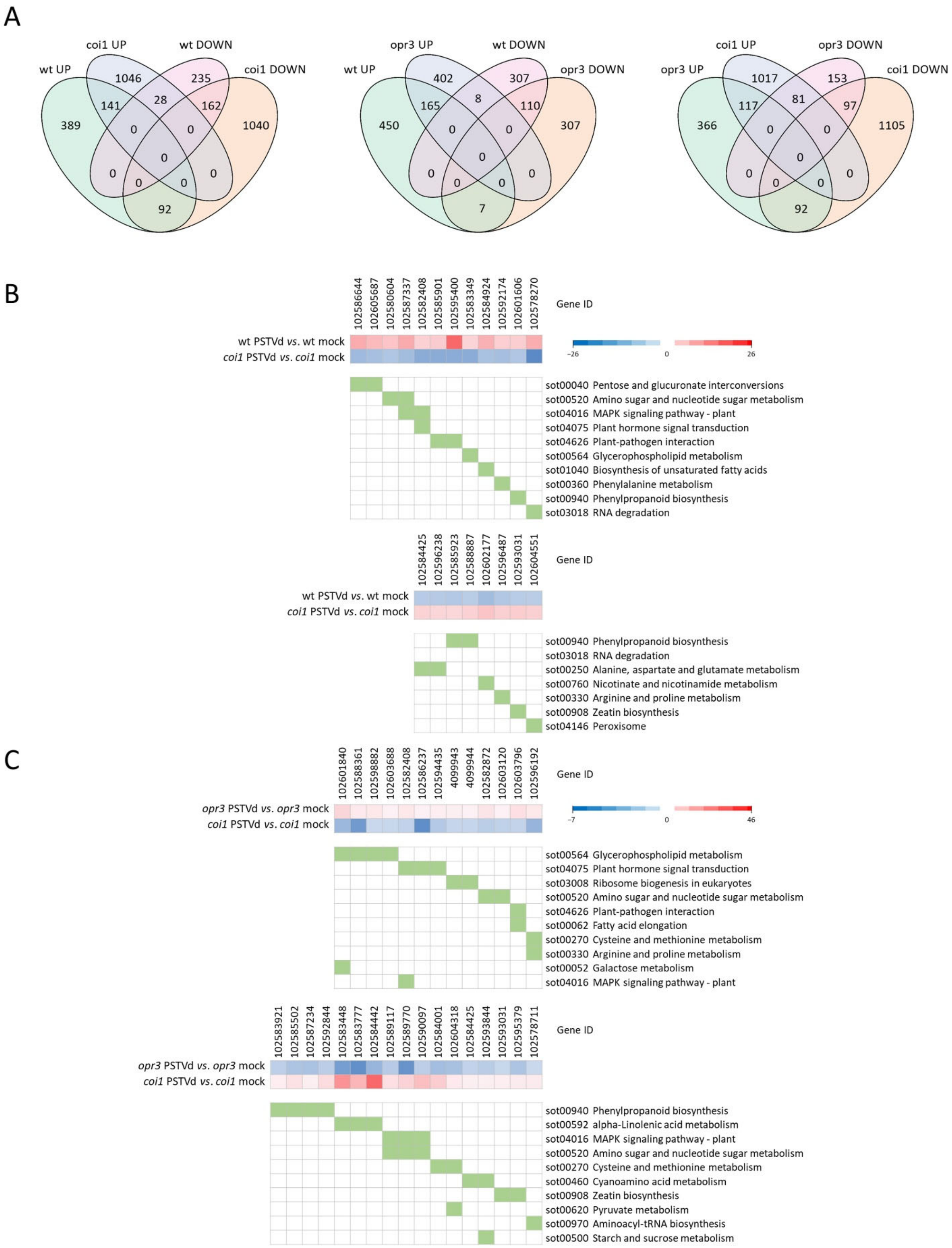

Figure S4. Analysis of DEGs with contrasting regulation between *opr3*, *coi1*, and wild-type potato plants infected with PSTVd. (**A**) Venn diagrams show overlaps between genes oppositely regulated in three different comparisons. DEGs from wt PSTVd *vs*. wt mock, *coi1* PSTVd *vs*. *coi1* mock, and *opr3* PSTVd *vs*. *opr3* mock comparisons are indicated by wt, *coi1*, and *opr3*, respectively. (**B**-**C**) Expression heatmaps of contrasting DEGs enriched in the top five KEGG pathways in the *coi1* vs. wt, and *coi1* vs. *opr3* comparisons. No enriched KEGG pathway was detected in the *opr3* vs. wt comparison. Gene expression values are shown as FPKM-normalized log2-transformed counts. Red and blue indicate upregulated and downregulated transcripts, respectively. Green square – enriched pathway; white square – not detected.

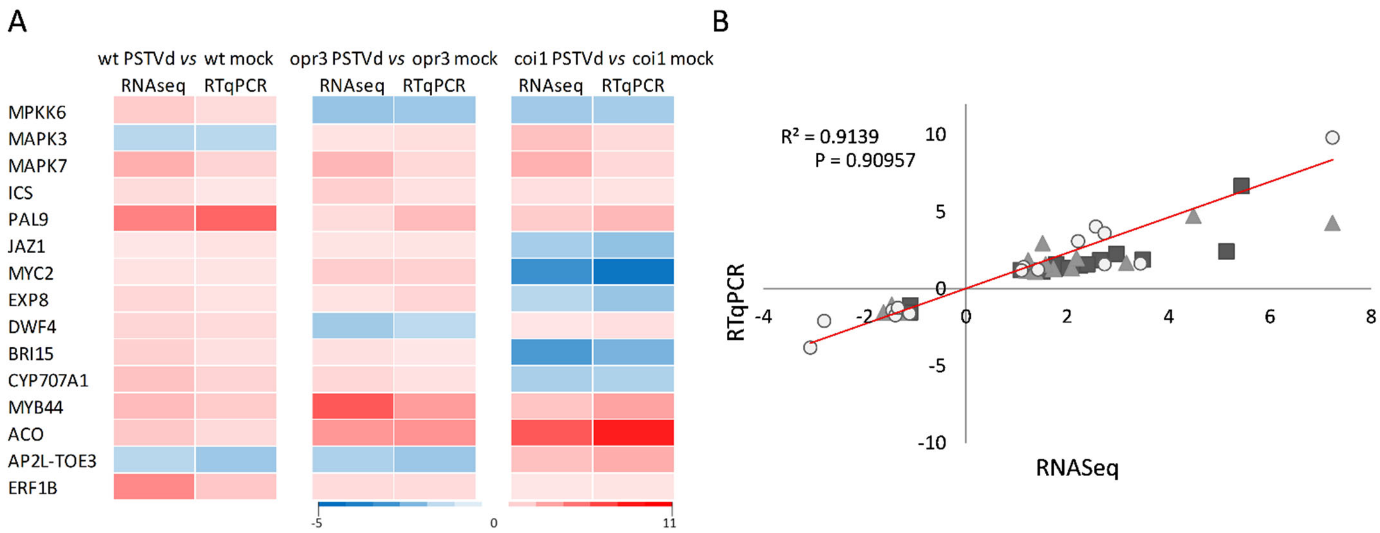

Figure S5. RT-qPCR validation of RNA-Seq results. (**A**) Heatmap of 15 selected DEGs in the three pairwise comparisons of PSTVd- and mock-inoculated plants. The color scale on heatmaps indicates the mean log2fc. (**B**) Correlation measured by log2fc method between RTqPCR (X-axis) and RNASeq (Y-axis). Squares correspond to the wt PSTVd vs. wt mock, triangles to the *opr3* PSTVd vs. *opr3* mock, and circles to the *coi1* PSTVd vs. *coi1* mock comparisons.

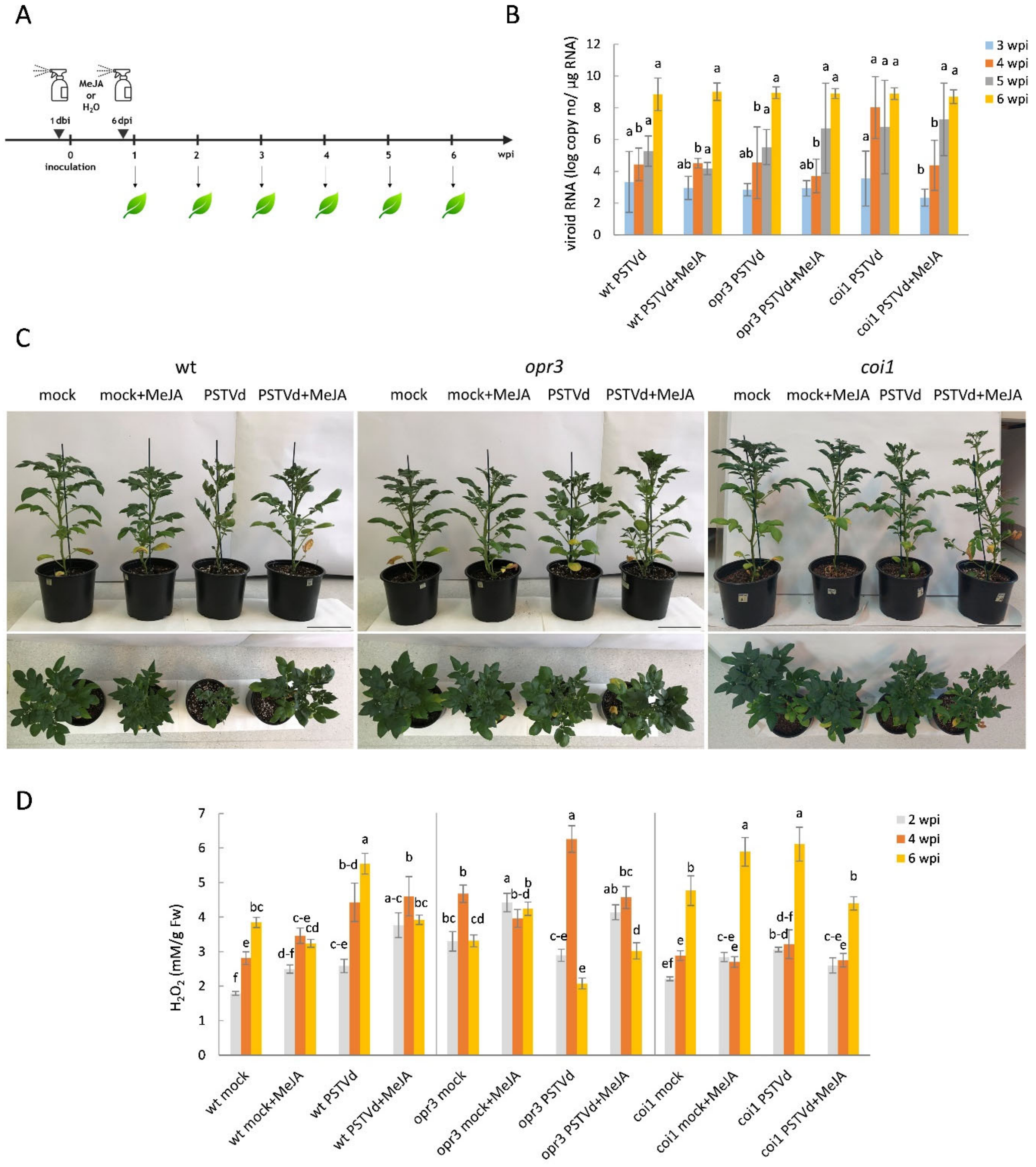

Figure S6. Effects of MeJA pretreatment on symptom development, H_2O_2 accumulation, and viroid RNA accumulation in *opr3*, *coi1*, and wild-type plants infected with PSTVd. (**A**) Graphical representation of the experimental design (dpi, days post inoculation; wpi, weeks post inoculation). (**B**) Viroid RNA was detected by absolute quantification using one-step RTqPCR. Values are means ± SE (n = 6) from one representative experiment. (**C**) Images show symptoms on plants at 6 wpi; bar = 15 cm. (**D**) H_2O_2 accumulation was determined spectrophotometrically at 2, 4, and 6 wpi. Values are means ± SE (n = 6) from one representative experiment. Different letters indicate significant differences among the three genotypes, assessed separately at each time point using DMRT ($p < 0.05$). Untreated mock plants (mock); MeJA-treated mock plants (mock+MeJA); PSTVd-infected untreated (PSTVd); MeJA-treated PSTVd-infected (PSTVd+MeJA).
